# Chronic companions: An updated national cross-sectional study of metabolic syndrome comorbidities in outpatient visits for hidradenitis suppurativa

**DOI:** 10.1371/journal.pone.0333010

**Published:** 2025-10-24

**Authors:** Elaine J. Ma, Alyssa M. Roberts, Peichi Chou, Abigail Katz, Charlotte Y. Jeong, Yvonne Nong, Maria T. Ochoa, April W. Armstrong

**Affiliations:** 1 University of Southern California Keck School of Medicine, Los Angeles, California, United States of America; 2 University of Hawaii at Manoa John A. Burns School of Medicine, Honolulu, Hawaii, United States of America; 3 University of California Riverside School of Medicine, Riverside, California, United States of America; 4 Icahn School of Medicine at Mount Sinai, New York, New York, United States of America; 5 University of Arkansas for Medical Sciences College of Medicine, Little Rock, Arkansas,; 6 Division of Dermatology, Department of Medicine, David Geffen School of Medicine at the University of California, Los Angeles, California, United States of America; 7 Division of Dermatology, Department of Medicine, University of Southern California Keck School of Medicine, Los Angeles, California, United States of America; Istituto Dermopatico dell'Immacolata (IDI)-IRCCS, ITALY

## Abstract

Hidradenitis suppurativa (HS) is a painful, chronic inflammatory skin disease associated with significant physical and psychosocial burden. Increasing evidence suggests HS is linked to systemic metabolic dysfunction, including components of metabolic syndrome such as obesity, hypertension, and hyperlipidemia. This study aimed to assess the prevalence of metabolic comorbidities in patients with HS using data from the National Ambulatory Medical Care Survey (NAMCS), a nationally representative dataset of U.S. outpatient visits from 2014 to 2019. We conducted a cross-sectional analysis comparing HS-related visits to age- and sex-matched non-HS visits, using multivariate logistic regression adjusted for demographic and clinical covariates. Among 1.8 million weighted HS-related visits, the most prevalent metabolic comorbidities were hypertension (15.7%), obesity (8.6%), and hyperlipidemia (7.4%). Compared to non-HS controls, HS visits had significantly higher odds of hypertension (adjusted odds ratio [aOR] 2.90; 95% confidence interval [CI]: 2.88–2.92), obesity (aOR 3.12; 3.10–3.15), and hyperlipidemia (aOR 1.76, 1.74–1.77). No significant association was found between HS and type 2 diabetes mellitus (T2DM) or cerebrovascular disease. Mechanistically, chronic systemic inflammation in HS, driven by elevated cytokines such as TNF-α, IL-6, and IL-17, may contribute to endothelial dysfunction and metabolic dysregulation. Obesity, which is commonly associated with HS, exacerbates the inflammatory state and promotes follicular occlusion, while hyperlipidemia may amplify inflammation through oxidative stress and impaired immune resolution. These findings underscore the importance of recognizing metabolic risk factors in patients with HS, particularly within the context of outpatient settings where early intervention is feasible. Early identification and management of these comorbidities may improve long-term health outcomes. Further longitudinal studies are warranted to clarify causal relationships and support the development of multidisciplinary screening and care strategies for this high-risk population.

## Introduction

Hidradenitis suppurativa (HS) is a chronic, relapsing inflammatory skin disorder characterized by recurrent painful nodules, abscesses, sinus tract formation, and scarring, predominantly affecting intertriginous areas such as the axillae, groin, and inframammary folds [1]. HS significantly impairs quality of life due to both physical symptoms and psychosocial distress and is estimated to affect up to 4% of the global population [2,3]. Despite its prevalence, the precise pathogenesis of HS remains incompletely understood. Current evidence suggests a multifactorial etiology involving genetic predisposition, follicular occlusion, dysregulated innate immunity, and environmental triggers such as smoking and obesity [4–5]. Additionally, emerging evidence suggests that epigenetic modifications, such as DNA methylation and histone modification, and dysregulation of the skin microbiome contribute meaningfully to the complex pathogenesis of HS, potentially influencing immune responses, inflammation, and skin barrier function [6–7].

Metabolic dysregulation, including insulin resistance, visceral adiposity, and chronic low-grade systemic inflammation, has been increasingly recognized as both a potential contributor to HS pathophysiology and a consequence of its persistent inflammatory state [5]. HS is characterized by elevated levels of proinflammatory cytokines such as tumor necrosis factor-alpha, IL-1β, IL-6, IL-8, and IL-17A, which are also key mediators in the pathogenesis of metabolic syndrome and cardiovascular disease [4]. These overlapping inflammatory pathways suggest a biological link between HS and metabolic comorbidities, raising important clinical concerns regarding long-term systemic health in affected individuals.

Prior epidemiologic studies have reported associations between HS and components of metabolic syndrome, including hypertension, dyslipidemia, obesity, and type 2 diabetes mellitus (T2DM).^9^ However, existing literature is often derived from single-center studies that may not capture the broader population-level burden of disease. In particular, there remains a lack of nationally representative data assessing the prevalence of these comorbidities in the outpatient setting, where most HS care is delivered and where opportunities for early risk identification and preventive care are most feasible.

Given the chronic and recurrent nature of HS and its frequent management in ambulatory care settings, evaluating comorbid conditions during outpatient visits provides meaningful insight into the real-world clinical landscape of HS care. Understanding how metabolic comorbidities present and are documented in these encounters can inform screening practices and multidisciplinary management strategies. To address this gap, our study examines the prevalence of metabolic syndrome-related comorbidities among patients with HS using data from a nationally representative survey of office-based outpatient visits in the United States.

## Methods

We conducted a cross-sectional, population-based study analyzing outpatient visits for patients with HS using data from the National Ambulatory Medical Care Survey (NAMCS) collected between 2014 and 2019. NAMCS is an annual survey administered by the Centers for Disease Control and Prevention (CDC) that provides nationally representative estimates of outpatient visits to non-federally employed, office-based physicians in the United States. The survey employs a complex multistage probability sampling design, including stratification and clustering, and uses masked weighting variables to generate population-level estimates. HS-related visits were identified using International Classification of Diseases codes: ICD-9 code 705.83 and ICD-10 code L73.2, corresponding to a clinical diagnosis of hidradenitis suppurativa. To evaluate the association between HS and metabolic comorbidities, we performed multivariate logistic regression analyses comparing HS-related visits to a control group of age- and sex-matched non-HS outpatient visits from the same dataset. The primary outcome variables were the presence of metabolic syndrome-associated comorbidities, including hypertension, obesity, hyperlipidemia, type 2 diabetes mellitus, and cerebrovascular disease documented during the visit. Regression models were adjusted for potential confounding factors including age, sex, race/ethnicity, insurance type, and tobacco use. Statistical significance was defined as a p-value less than 0.05. Data management and statistical analyses were performed using SAS Studio version 3.82.

## Results

A total of 1,800,930 weighted outpatient visits for patients with HS were included in the analysis. The majority of patients were female (62.9%), with males comprising 37.1% of visits. The racial and ethnic distribution was 73.2% White, 8.5% Black, 11.1% Hispanic, and 7.2% non-Hispanic other. Regarding insurance status, 58.1% of visits were covered by private insurance, 20.8% by Medicaid, the Children’s Health Insurance Program (CHIP), or other state-based programs, 12.8% by Medicare, and 1.5% were self-pay. Current tobacco use was reported in 25.0% of HS visits, while 52.1% of HS visits reported previous tobacco use (Fig 3).

**Fig 1:**
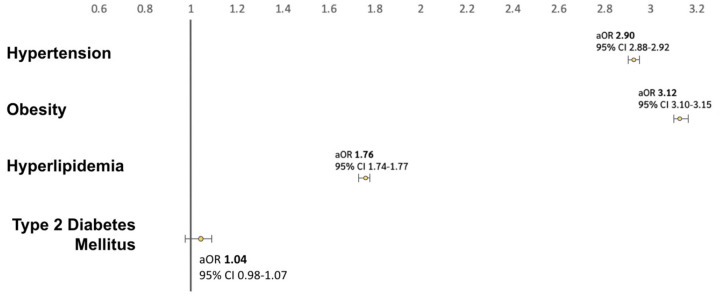
Multivariate logistic regression analysis of the association between metabolic comorbidities and HS patient visits compared to age- and sex-matched non-HS patient visits after adjusting for age, sex, race/ethnicity, insurance type, and tobacco use.

**Fig 2:**
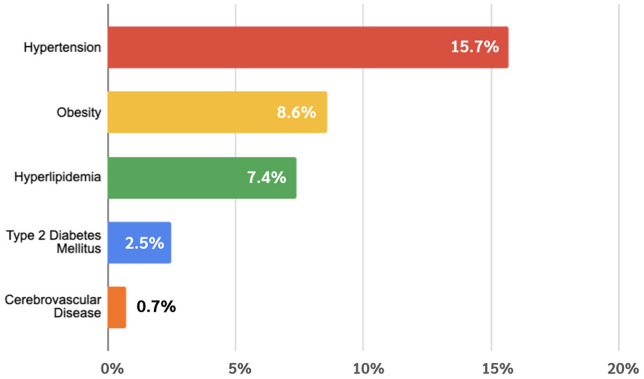
Prevalence of metabolic comorbidities in HS outpatient visits.

The prevalence of metabolic comorbidities among HS visits was as follows: hypertension (15.7%), obesity (8.6%), hyperlipidemia (7.4%), T2DM (2.5%), and cerebrovascular disease (0.7%) (Fig 2). Compared to 8,755,033 age- and sex-matched non-HS control visits, multivariate logistic regression demonstrated significantly higher odds of metabolic comorbidities among HS patients. After adjusting for age, sex, race/ethnicity, insurance type, and tobacco use, HS visits had nearly three times the odds of hypertension (adjusted odds ratio [aOR] 2.90; 95% confidence interval [CI]: 2.88–2.92), and more than three times the odds of obesity (aOR 3.12; 95% CI: 3.10–3.15). The odds of hyperlipidemia were also elevated (aOR 1.76; 95% CI: 1.74–1.77) (Fig 1).

In contrast, there was no significant association between HS and either T2DM or cerebrovascular disease. This may be due to the relatively low prevalence of these conditions within the surveyed population where the mean age was 32.1 years (standard error of the mean: 0.9), younger than the typical age of onset for both conditions which result from the cumulative impact of metabolic dysfunction.

## Discussion

This cross‐sectional study characterizes patterns of metabolic comorbidities associated with HS through age- and sex-matched comparisons using a nationally representative sample of U.S. outpatient visits. Our analysis reveals significantly higher odds of hypertension, hyperlipidemia, and obesity among patients with HS compared to matched non-HS controls, reinforcing the growing recognition of HS as a systemic inflammatory condition with substantial metabolic burden. These findings align with prior studies suggesting an elevated cardiometabolic risk profile in individuals with HS [9].

Growing evidence points to a compelling mechanistic link between HS and hypertension, rooted in systemic inflammation, metabolic dysregulation, and vascular dysfunction. HS is characterized by the chronic release of pro-inflammatory cytokines such as TNF‑α, IL‑1β, IL‑17, and IL‑23 from active skin lesions.^1^ These inflammatory mediators circulate systemically, contributing to endothelial dysfunction and arterial stiffness, two key drivers of hypertension. In particular, cytokines like IL‑6 and CRP, which are elevated in HS, have been implicated in vascular inflammation and remodeling, leading to increased vascular resistance and blood pressure [10–11].

Adipose tissue in individuals with obesity functions as an active endocrine organ, releasing pro-inflammatory cytokines such as TNF‑α, IL‑6, and leptin, which amplify systemic inflammation and may worsen HS disease severity [4,14]. Obesity also contributes to mechanical stress in intertriginous areas, promoting follicular occlusion and lesion formation [8]. Additionally, the chronic low-grade inflammation associated with obesity may perpetuate the immune dysregulation seen in HS, including increased Th17 signaling and neutrophil recruitment [10–11]. Systemic inflammation in HS can alter lipid metabolism, promoting hepatic overproduction of triglyceride-rich lipoproteins and reducing clearance of LDL particles [12]. Additionally, elevated levels of pro-inflammatory cytokines such as TNF-α and IL-6 may impair lipid regulation and contribute to dyslipidemia. HS is also associated with obesity, which further disrupts lipid homeostasis and increases circulating levels of atherogenic lipoproteins. Oxidized LDL can stimulate endothelial activation and monocyte recruitment, while elevated triglycerides are linked to small, dense LDL particles and increased inflammatory lipoproteins [12–13].

The cross-sectional, visit-based design of this large population study may limit its ability to fully capture the chronic comorbidities present in patients with HS. The observed prevalence of T2DM and cerebrovascular disease was unexpectedly low among HS patients in our sample. While prior research has established associations between HS and T2DM [14–15], our findings may reflect limitations inherent to the data source. NAMCS data is visit-based and includes only diagnoses addressed or documented during the clinical encounter; certain conditions may be underreported, particularly during visits focused on dermatologic concerns.

**Fig 3:**
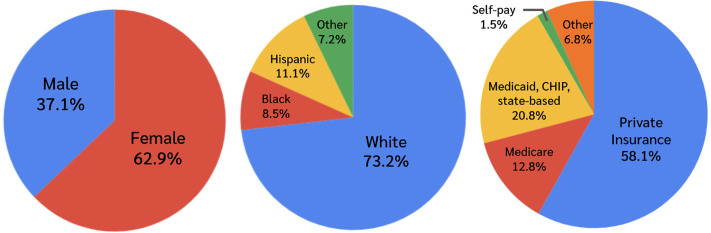
Sex, race, and insurance demographics of HS outpatient visits.

Despite these limitations, our findings highlight a critical opportunity for clinical awareness and intervention. HS patients, many of whom are young, may harbor undiagnosed components of metabolic syndrome [16]. Early recognition of this risk profile is essential, and our results support the need for targeted metabolic screening in patients presenting with HS, even in dermatologic settings Figs 1–3.

This study also underscores the need for more robust, longitudinal patient-level research to fully elucidate the relationship between HS and metabolic comorbidities. Future investigations should incorporate HS disease severity, which was not captured in the NAMCS dataset but likely to influence documentation and comorbidity burden. In addition, future research should include racially and ethnically diverse populations to better capture the full spectrum of HS burden and outcomes. While our findings offer valuable insights into the outpatient landscape of HS care, further prospective studies are needed to validate these associations and inform guidelines for comprehensive, multidisciplinary management of this high-risk population.
